# Identifying potential zones for rainwater harvesting interventions for sustainable intensification in the semi-arid tropics

**DOI:** 10.1038/s41598-022-07847-4

**Published:** 2022-03-10

**Authors:** Kaushal K. Garg, Venkataradha Akuraju, K. H. Anantha, Ramesh Singh, Anthony M. Whitbread, Sreenath Dixit

**Affiliations:** International Crops Research Institute for the Semi-Arid Tropics, Patancheru, Telangana 502324 India

**Keywords:** Environmental sciences, Hydrology

## Abstract

Decentralized rainwater harvesting (RWH) is a promising approach to mitigate drought in the drylands. However, an insufficient understanding of its impact on hydrological processes has resulted in poor resource planning in this area. This study is a meta-analysis of 25 agricultural watersheds representing a range of rainfall and soil types in the semi-arid tropics. Rainfall-runoff-soil loss relationship was calculated at daily, monthly and yearly levels, and the impact of RWH interventions on surface runoff and soil loss was quantified. A linear relationship was observed between daily rainfall and surface runoff up to 120 mm of rainfall intensity, which subsequently saw an exponential increase. About 200–300 mm of cumulative rainfall is the threshold to initiate surface runoff in the Indian semi-arid tropics. Rainwater harvesting was effective in terms of enhancing groundwater availability (2.6–6.9 m), crop intensification (40–100%) and farmers’ incomes (50–200%) in different benchmark watersheds. An average of 40 mm of surface runoff was harvested annually and it reduced soil loss by 70% (3 ton/ha/year compared to 1 ton/ha/year in non-intervention stage. The study further quantified runoff at 25th, 50th and 75th percentiles, and found that more than 70% of the area in the Indian semi-arid tropics has high to medium potential for implementing RWH interventions.

## Introduction

Globally, the drylands face a number of challenges such as water scarcity, land degradation, poor agriculture and livestock productivity^1–3^. With increasing population pressure, changing food habits, and economic development, per capita resource availability has been declining rapidly^4^. The global population is expected to reach 9.5 billion by 2050^5^. However, natural resources such as water and land will remain static as it is not possible to exploit these finite resources further^6–8^. Changing climatic conditions are adding to resource scarcity and uncertainty^9^. A number of studies have reported on the large untapped potential for sustainable crop intensification in the drylands as current land and water use efficiencies are much below achievable potential^10^. This has led to the implementation of a number of public welfare programs in Asia and Africa focusing on a range of in-situ conservation and ex-situ rainwater harvesting interventions (together referred to as rainwater harvesting in this study). More than US$ 2 billion per year is being invested on RWH interventions in India to mitigate climate related risks^11^. However, a lacuna in data on biophysical, hydrological, and land use and resource utilization patterns^12,13^ have resulted in the poor design and execution of RWH interventions, leading to the non-realization of their full potential.

Surface runoff is one of the critical water balance components taken into account while designing and implementing RWH interventions. However, there is a gap in understanding its response to rainfall variability^14^. Most water balance studies in the semi-arid tropics have been conducted at river basin scale or are based on simulation modeling^12,13^, with their outcomes difficult to scale down to field and meso scales (0.1 to 10 km^2^) for decision making at landscape/community scale. Similarly, soil loss and surface runoff are less understood. Most public welfare programs are focused on controlling land degradation and soil loss^15^. In the absence of measured data, it is difficult to realize the impact of RWH interventions that may require corrective measures for refinements.

**Figure 1:**
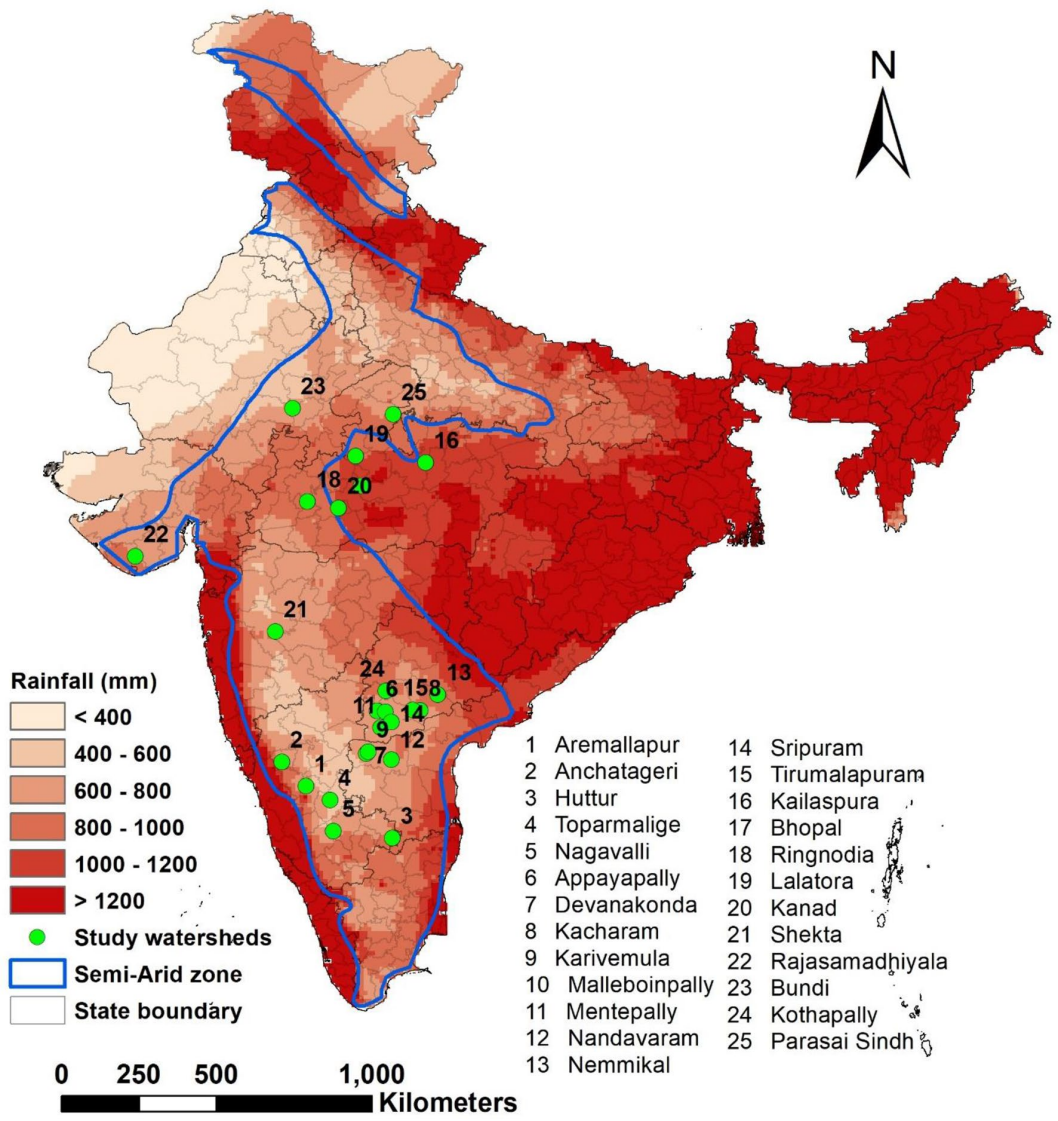
Location of 25 watersheds studied across different rainfall regions in the Indian semi-arid tropics. Watershed numbers 4, 12, 13, 16, 17, 18, 19, 20, 21 and 24 had Vertisols while the rest had Alfisols.

To bridge this gap, the International Crops Research Institute for the Semi-Arid Tropics (ICRISAT) carried out a number of research and development initiatives between 1999 and 2016. This study is a meta-analysis of 25 of these case studies conducted in the semi-arid tropics (rainfall between 400 and 1200 mm), wherein primary data collected from field measurements was used (Fig. 1). The objectives of the paper are to: (i) establish rainfall-runoff-soil loss relationships; (ii) analyse the impact of RWH interventions on surface runoff, soil loss and other ecosystem services; and (iii) quantify runoff probabilities and identify potential zones to implement RWH interventions in the semi-arid tropics of the Indian subcontinent.

## Materials and methods

### Study watersheds

**Figure 2:**
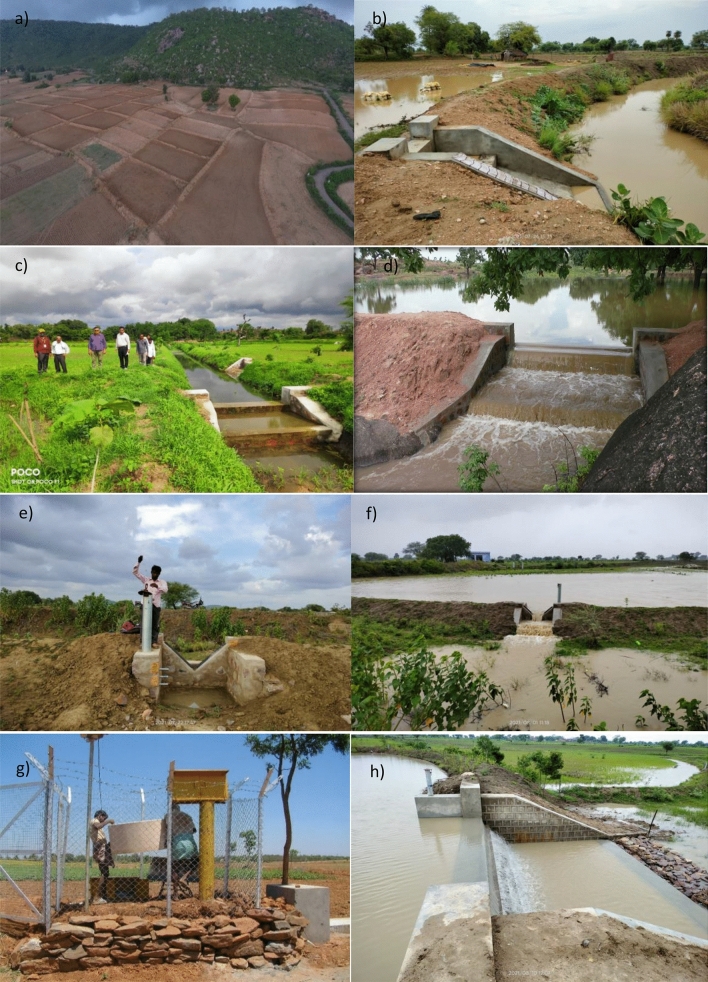
Different methods of rainwater harvesting and the hydrological monitoring system set up in the study watersheds: (**a**, **b**) In-situ and (**c**, **d**) ex-situ rainwater harvesting interventions and (**e**–**h**) hydrological monitoring gauges to estimate surface runoff and soil loss.

In the last two decades, ICRISAT together with its consortium partners has implemented a number of research and development initiatives focusing on natural resource management ^15–35^. Figure 1 shows the watersheds studied in the semi-arid tropics, where annual average rainfall varies between 400 and 1200 mm. Groundwater is the primary source of freshwater to meet domestic and agricultural demand and is generally in short supply after the monsoon season. Agricultural productivity in these watersheds before the project interventions ranged between 0.5 and 2.0 ton/ha. Nearly 30–40% of agricultural land remained fallow largely due to water scarcity^18,19^. More than 90% of the farmers in these watersheds owned less than 2.0 ha, were poor and endured malnutrition^18,19^. Given these conditions, the watersheds were developed as sites of learning by implementing a range of in-situ conservation and ex-situ rainwater harvesting interventions at landscape and farm scales (Fig. 2).

Earthen field bunds, retention ditches, and raised beds were the common in-situ interventions implemented mainly in the degraded uplands to enhance residual soil moisture and control land degradation^19^. A ridge-to-valley approach was followed exposing upland locations to in-situ interventions to protect the landscape from excessive erosion to enhance water retention ability through earthen structures and biological measures. Fields of 1–2 ha were divided into 6–8 parcels with earthen bunds (the cross-section ranging between 0.60 m^2^ and 1.2 m^2^) with appropriate field drainage masonry structures to dispose of excess runoff to downstream fields. Such interventions reduce the velocity of the flowing water and provide an opportunity to harvest a fraction of the surface runoff, thereby helping enhance soil moisture availability and groundwater recharge^15^ (refer Fig. 2a,b).

In addition, *ex-situ* interventions such as the construction of masonry check dams, desilting and renovation of traditional water bodies and farm/community ponds was undertaken in a decentralized manner on 10–500 ha of catchment with rainwater harvesting capacity of 5000–50,000 m^3^ ^17–19^ (Fig. 2c,d). These interventions help control excessive erosion and stabilize the landscape. The structures also harvest surface runoff on individual or community land^36–38^ and enhance surface and groundwater availability for multiple purposes in the rural community.

Table 1 shows the direct outreach of these initiatives in 75 villages covering 29,500 ha and involving about 9500 farming households. Of the total geographical area, about 10% of the landscape, especially uplands, were treated with in-situ conservation measures and about 45% of the area with ex-situ rainwater harvesting interventions. These interventions created about 3.0 Million Cubic Meters (MCM) of rainwater harvesting storage capacity.

**Table 1:** Summary of demographic and project interventions undertaken in study watersheds between 1999 and 2016.

Indicators	Mean	Min	Max	Total
Number of villages (nos)	3	1	14	75
Number of households (nos)	430	170	1400	9500
Population (nos)	2160	855	7000	47,500
Geographical area (ha)	1280	390	4800	29,500
Project duration (years)	4	2	6	–
Area treated with RWH interventions (ha)	600	80	1500	13,000
Total area treated with in-situ interventions (ha)	160	5	790	3400
Water storage capacity created (MCM)		0.003	1.5	3.0
Period of hydrological monitoring (years)	3	1	7	–

### Data collection and analysis

**Figure 3:**
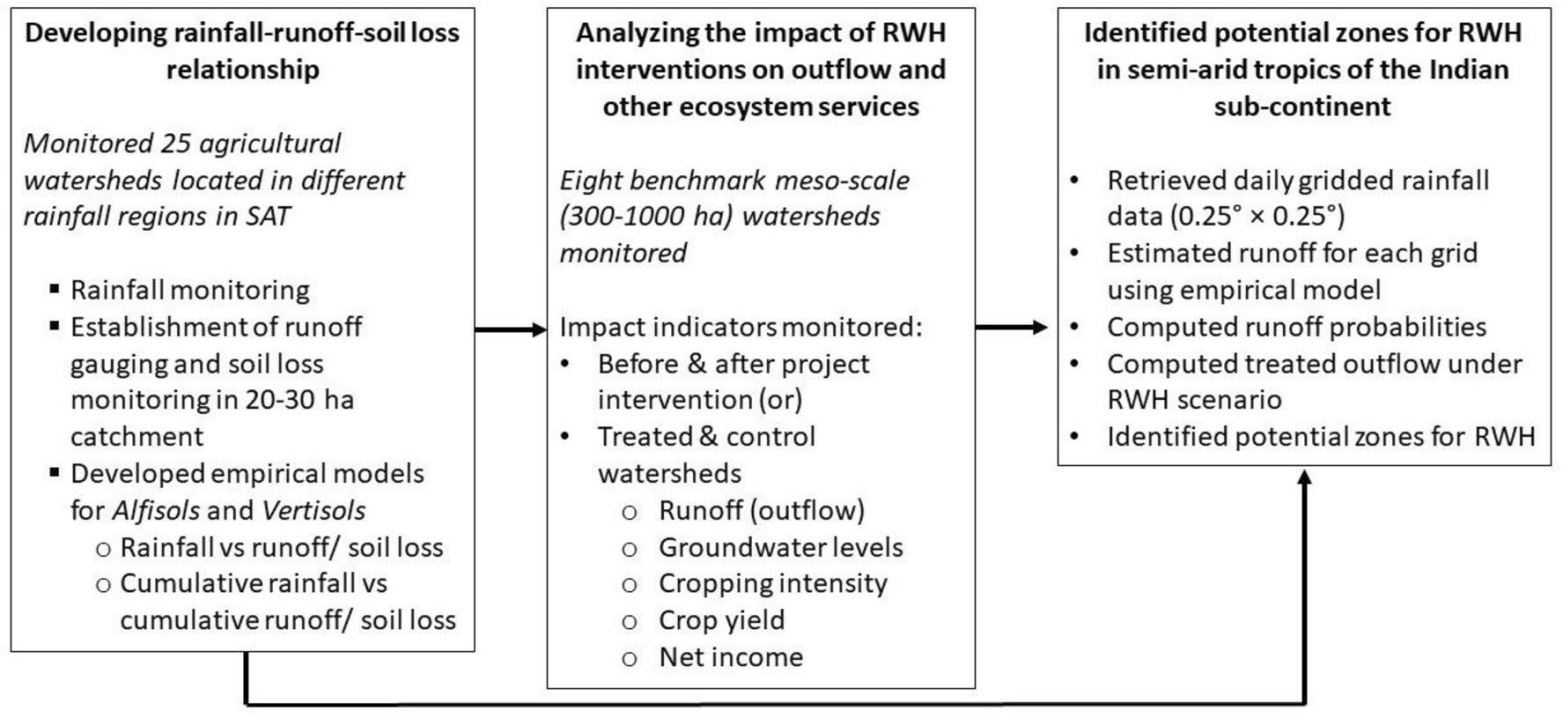
Description of the methodology and data used.

Figure 3 outlines the methodology followed in the study. The study analysed data collected from 25 agricultural watersheds which were monitored in terms of surface runoff and soil loss. Of these, 8 benchmark watersheds were monitored intensively for groundwater levels, change in land use, cropping intensity, and crop yields under both treated and control conditions. While a treated watershed is a landscape with RWH interventions in place, a control watershed is the untreated landscape nearby. Based on the outcomes of the primary data, empirical models were developed to estimate surface runoff and applied to the semi-arid tropics of the Indian subcontinent to estimate runoff potential at different probabilities. A detailed description of the data collection and methodology adopted in the current study follows.

#### Monitoring rainfall, runoff and soil loss

Rainfall was monitored through meteorological stations set up in the pilot watersheds. Automatic rain gauges were installed at the pilot watersheds to monitor rainfall on an hourly basis. Runoff was monitored on 20–30 ha of the catchment area in 23 of the 25 intervention sites. Of these, 15 watersheds had light textured Alfisols and 10 watersheds had heavy textured Vertisols (Table 2). Runoff recording was intensified on 300–1000 ha in select watersheds (#19, 21, 22, 23, 24, 25, refer Fig. 1) by following the paired watershed (treated and control watersheds) approach.

**Table 2:** Average annual rainfall, runoff and soil loss measured along with peak intensity events in different pilot watersheds in the semi-arid tropics.

w/s no	District	Name of the watershed	Soil group	Clay (%)	Silt (%)	Sand (%)	Gravel (%)	FC (g/g)	PWP (g/g)	OC (%)	Avg. rainfall (mm)	Avg. runoff (mm)	Avg. runoff coefficient (−)	Soil loss (t/ha)	Years of monitoring	Peak intensity rainfall (mm)	Peak runoff (mm)	Peak soil loss (t/ha)
1	Haveri	Aremallapur	Alfisols	15.19	18.99	65.82	58.00	0.15	0.09	0.51	458	44	9.6	2.03	3	177	71	3.43
2	Dharwad	Anchatageri	Alfisols	22.22	22.23	55.55	8.57	0.20	0.13	0.65	563	44	7.8	1.53	4	210	54	2.60
3	Kolar	Huttur	Alfisols	14.71	11.53	73.76	1.38	0.11	0.07	0.38	567	30	5.3	0.91	3	61	10	0.49
5	Tumkur	Nagavalli	Alfisols	8.02	6.88	85.10	0.04	0.03	0.09	0.39	550	40	7.3	–	3	70	24	–
6	Mahbubnagar	Appayapally	Alfisols	–	–	–	–	–	–	0.34	568	51	9.0	0.38	2	44	13	0.20
7	Kurnool	Devanakonda	Alfisols	–	–	–	–	–	–	0.38	534	41	7.7	0.31	2	105	21	0.22
8	Nalgonda	Kacharam	Alfisols	–	–	–	–	–	–	0.42	702	18	2.6	0.19	2	43	10	0.14
9	Kurnool	Karivemula	Alfisols	–	–	–	–	–	–	0.42	320	20	6.3	0.57	1	66	16	0.53
10	Mahbubnagar	Malleboinpally	Alfisols	–	–	–	–	–	–	0.37	654	34	5.2	0.00	1	45	24	–
11	Mahbubnagar	Mentapally	Alfisols	–	–	–	–	–	–	0.35	335	20	6.0	0.20	1	38	10	0.10
14	Mahbubnagar	Sripuram	Alfisols	–	–	–	–	–	–	0.40	440	19	4.3	0.29	2	28	14	0.23
15	Nalgonda	Tirumalapuram	Alfisols	–	–	–	–	–	–	0.42	474	11	2.3	0.35	1	25	5	–
22	Rajkot	Rajasamadhiyala	Alfisols	19.30	27.50	53.20	1.2	0.30	0.20	0.81	550	–	–	–	–	–	–	–
23	Bundi	Bundi	Alfisols	14.00	30.00	56.00	9.00	0.19	0.10	0.40	439	63	14.4	2.11	5	121	70	2.72
25	Jhansi	Parasai-sindh	Alfisols	18	25	57	5.0	0.15	0.05	0.60	720	136	18.9	–	5	90	40	–
4	Chitradurga	Toparmalige	Vertisols	52.07	22.94	24.99	5.00	0.36	0.22	0.6	447	34	7.6	1.54	3	170	60	2.5
12	Kurnool	Nandavarm	Vertisols	–	–	–	–	–	–	0.53	427	15	3.5	0.1	1	30	5	0.05
13	Nalgonda	Nemmikal	Vertisols	–	–	–	–	–	–	0.41	695	46	6.6	0.89	1	90	16	0.62
16	Guna	Kailaspura	Vertisols	45.20	30.70	24.00	1.70	0.30	0.16	0.65	866	195	22.5	4.09	4	219	139	2.89
17	Bhopal	IISS, Bhopal	Vertisols	45.36	31.18	23.46	0.00	0.30	0.17	0.54	923	135	14.6	1.10	3	163	54	1.85
18	Indore	Ringnodia	Vertisols	–	–	–	–	–	–	0.70	794	98	12.3	–	6	80	32	–
19	Vidisha	Lalatora	Vertisols	47.23	31.02	21.75	2.60	0.25	0.18	0.54	958	234	24.4	3.10	1	103	72	0.96
20	Devas	Kanad	Vertisols	–	–	–	–	–	–	1.3	743	166	22.3	7.40	3	247	224	10.30
21	Ahmednagar	Sekta	Vertisols	–	–	–	–	–	–	0.50	561	–	–	–	–	–	–	–
24	Medak	Kothapally	Vertisols	62.79	19.75	17.47	6.60	0.40	0.20	1.04	756	85	11.2	2.82	7	128	80	3.80

Level of significance																		
Mean			Alfisols	15.92	20.30	63.78	11.88	0.16	0.10	0.45	524	41	7.6	0.7	2.5	80	27	1.1
Mean			Vertisols	50.5	27.1	22.3	3.18	0.32	0.19	0.68	717	112	13.9	2.6	3.2	137	76	2.9
F value											9.57	9.96		7.00	6.23	4.61	6.18	2.60
F critical											4.30	4.32		4.41	4.32	4.325	4.325	4.494
p-value											0.01	0.00		0.02	0.02	0.044	0.021	0.126
Significance level (p < 0.05)											Significant	Significant		Significant	Significant	Significant	Significant	Not significant

*FC* Field capacity, *PWP* Permanent wilting point, *OC* Organic carbon.

To establish runoff gauging stations and monitor soil loss, H-flume, V-notch (Fig. 2e,f) or rectangular weirs (Fig. 2g,h) with masonry structures were constructed at the outlets. A stage recorder coupled with a soil monitoring system were installed at the gauging stations^18^. The runoff devices were programmed to capture water level (stage) in the stilling well at 2–10-min intervals depending on catchment size. The device does smart sampling by linking runoff sampled to sediment load^19,39,40^. During runoff, water flowing at hourly intervals was pumped automatically and stored in separate containers (Fig. 2g). To measure soil loss, these water samples were analyzed in a laboratory for sediment concentration. The hydrograph of each runoff event was divided into 60-min time segments and sediment concentration data was superimposed on it to estimate soil loss^19^. This was computed by multiplying the volume of segment runoff by sediment concentration^39,40^. Equations (1) and (2) were used to estimate surface runoff and Eq. (3) was used to calculate soil loss in different runoff events.

1 $${\text{Discharge}} \;Q_{t} \left( {\frac{{m^{3} }}{s}} \right) = 1.705L \times \left( {h_{t} } \right)^{1.5}$$

where, L = length of the rectangular weir; h_t_ = depth of runoff layer passing from gauging station at a given time (t).

2 $$Discharge \, volume \left({m}^{3}\right)= \sum_{i=1}^{t=n}Discharge \, rate \left(\frac{{m}^{3}}{\mathrm{Sec}}\right) \times time \, interval (s)$$

3 $$Soil \, loss \left(kg\right)=\sum_{t=1}^{t=n}Runoff \, volume \, \left(\frac{{m}^{3}}{\mathrm{s}}\right) \times sediment \, concentration \left(\frac{Kg}{{m}^{3}}\right)$$

All the event hydrographs were analysed to estimate runoff against different rainfall events, and rainfall-runoff-soil loss relationships were established on a daily, monthly and yearly basis. Runoff coefficient was estimated for different months in the monsoon season and statistical test, analysis of variance (ANOVA) were performed to understand the level of significance. Further, post hoc tests were conducted to identify the significance for different months. In addition, T-test was performed to understand the difference in runoff coefficients among the Vertisols and Alfisols watersheds.

Outflow (i.e., surplus runoff from the watershed outlet) measured from treated and control watersheds were compared and reduction in surface runoff due to RWH (referred to as harvesting threshold) was estimated.

#### Groundwater table and cropping intensity

The groundwater table in dug wells was monitored in eight benchmark watersheds (# 16, 18, 19, 21, 22, 23, 24, 25). Nearly 50–300 wells per site were monitored from pre-project stage to post intervention using water level indicators on a monthly basis^17,19,20^. In addition, the number of pumping hours and area irrigated during rainy and postrainy seasons were monitored. The change in land use due to RWH interventions was mapped through household surveys and ground data collection. The area under agriculture and fallow lands was mapped for both rainy and postrainy seasons before and after project intervention.

#### Crop yield, cost of cultivation and income

Changes in land use and cropping intensity due to RWH interventions were captured through ground surveys before and after project implementation. In addition, about 2000 crop cutting studies (80–100 per site per season) were undertaken to assess the impact of best management practices on crop yield^41,42^. An area of 3 m × 3 m was demarcated with three replications. Crop was harvested at maturity to measure grain yield. In addition, cost of cultivation (farm inputs, irrigation, labour, energy) data was collected through household surveys of selected farmers before and after the interventions and net income was calculated^42,43^.

Yields of different crops (e.g., cotton, maize, groundnut, chickpea, wheat) grown by about 200 farmers in each watershed were assessed, covering both treated and control fields. The cost of cultivation including labour (counting own labour), seed, fertilizer, pesticides, irrigation and hiring of machinery was captured. Net income from agriculture constituted that from both rainy and postrainy seasons. Total gross income was obtained by multiplying crop yield (obtained from crop cutting studies) with market price and subtracted the total cost of cultivation. Inflation was taken into account while estimating the cost of cultivation and net income. Net income generated before and after project interventions were estimated as per Eq. (4).

4 $$NI_{a} = \sum\limits_{{i = 1}}^{n} {Y_{i} \times A_{i} - C_{i} }$$

where, NI_a_ = Net income (US$/ha/year); Y_i_ = crop yield (t/ha) for plot i; A_i_ = area of the plot i (ha); C_i_ = cost of cultivation of plot i (US$/ha); and n = number of plots farmers own.

### Analyzing runoff probabilities and identifying potential zones for rainwater harvesting

Daily gridded rainfall data between the years 1985 and 2018 with a resolution of 0.25° × 0.25° was sought from the India Meteorological Department, Pune, Government of India^44^. Time series data was available for 4906 grid points covering all of India. However, this analysis only focused on semi-arid regions of India covering 128.4 Million ha; hence 1799 grid points were selected (Fig. 1). Empirical rainfall-runoff relationship developed from 25 watersheds was used to estimate surface runoff using IMD’s daily rainfall data. The estimated runoff events were aggregated annually for each of the grid points. Rainfall of less than 2.5 mm/day was excluded from the analysis as empirical analysis shows negligible runoff below this threshold. Average annual runoff estimated for 33 years was arranged in chronological order for each grid and runoff amounts for the 25th, 50th, and 75th percentiles were retrieved from time series of the respective grid points. These runoff values were interpolated using Inverse Distance Weighting (IDW)^45^, wherein interpolated values for each location were computed from the weighted sum of the grid points.

A RWH scenario was created to understand the surplus runoff (i.e., outflow) after the landscape treatment, as per Eq. (5):

5 $${\text{Outflow}}_{{{\text{treated}} - {\text{i}}}} = {\text{ Runoff }}\,{\text{at}}\,{ 5}0{\text{th}}\,{\text{percentile }}\,\left( {{\text{mm}}} \right)_{{{\text{control}} - {\text{i}}}} {-}{\text{ harvesting}}\,{\text{ threshold}} \, \left( {{\text{mm}}} \right)$$

Further, potential zones for rainwater harvesting were mapped as per the decision rule below:

- If Outflow _treated−i_ > 0.70 × runoff, it is a high potential zone;
- If Outflow _treated−i_ ≤ 0.70 × runoff and ≥ 0.50 × runoff; it is a medium potential zone; and.
- If Outflow _treated−i_ < 0.50 × runoff, it is a low potential zone.

## Results

### Rainfall characterization

**Figure 4:**
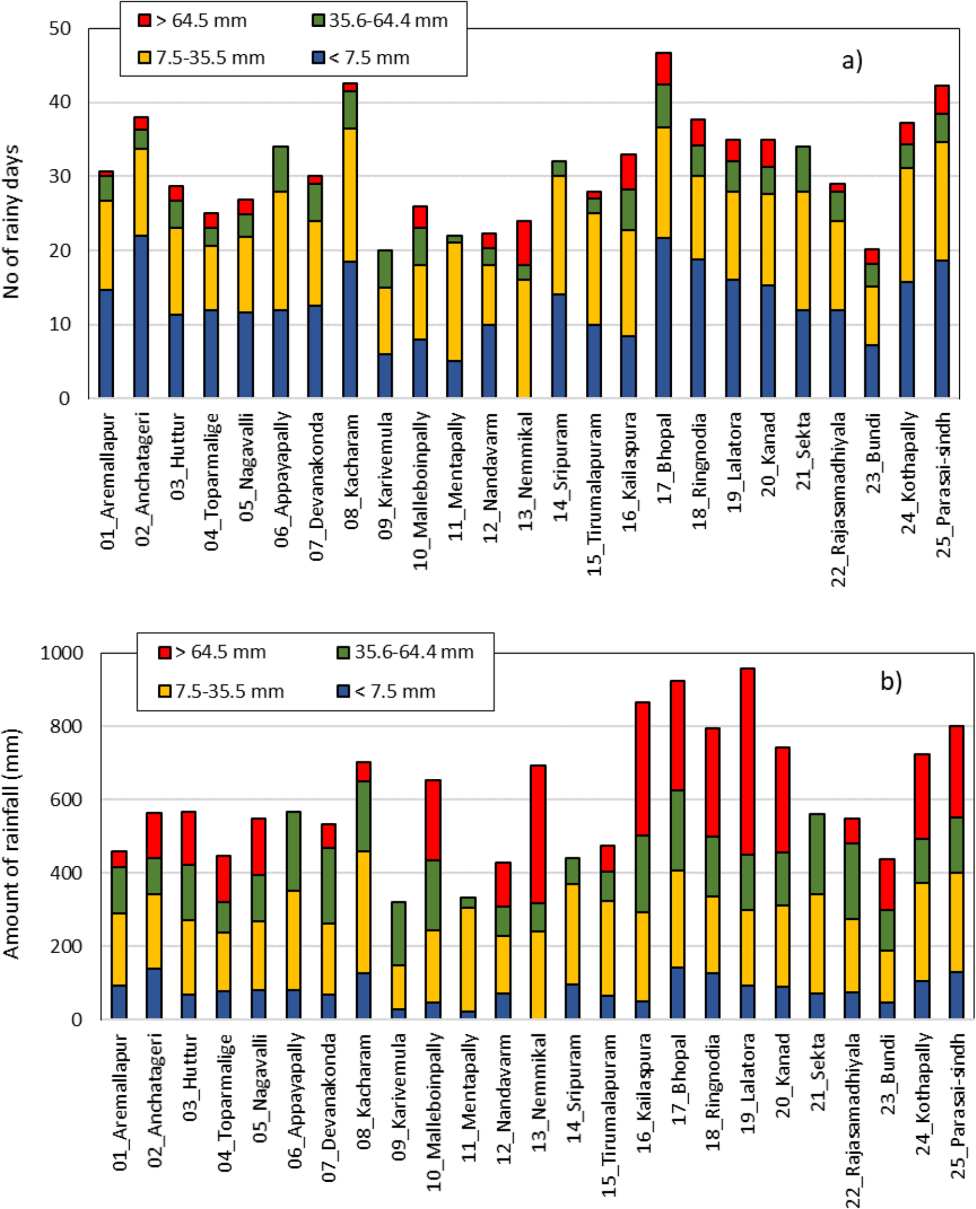
Distribution of rainfall in the study watersheds.

Figure 4a shows the number of rainy days and Fig. 4b the amount of rainfall from different intensity events in a year in the study watersheds during the monitoring period. Daily rainfall events (mm/day) were categorized into four categories: light intensity rainfall with < 7.5 mm/day; moderate intensity with 7.5–35.5 mm/day; high intensity with 35.6–64.4 mm/day; and very high intensity with > 64.5 mm, as per the IMD classification^46^. The average number of rainy days in the study watersheds was 31, ranging between 20 and 47 days. Of the 25 watersheds, 22 received less than 40 days of rainfall in a year during the monitoring period. Out of 31 rainy days, 25 days saw low to moderate intensity rainfall events and only 6 days received high and very high intensity rainfall on an average. However, rainfall intensity varied with location (Fig. 4a).

In these locations, average annual rainfall of 604 mm was recorded, ranging between 320 and 958 mm. Of the total annual rainfall received, 80 mm (13%) came from low intensity events, 223 mm (37%) from moderate intensity events, and 301 mm (50%) from high and very high intensity events (Fig. 4b). It may be noted that about 50% of the annual rainfall was received in 6 days from high to very high intensity rainfall events on an average. Among the watersheds, Karivemula (# 9) and Mentapally (# 11) received minimal rainfall of 320–335 mm and Lalatora (# 19) received the highest amount of 958 mm.

**Figure 5:**
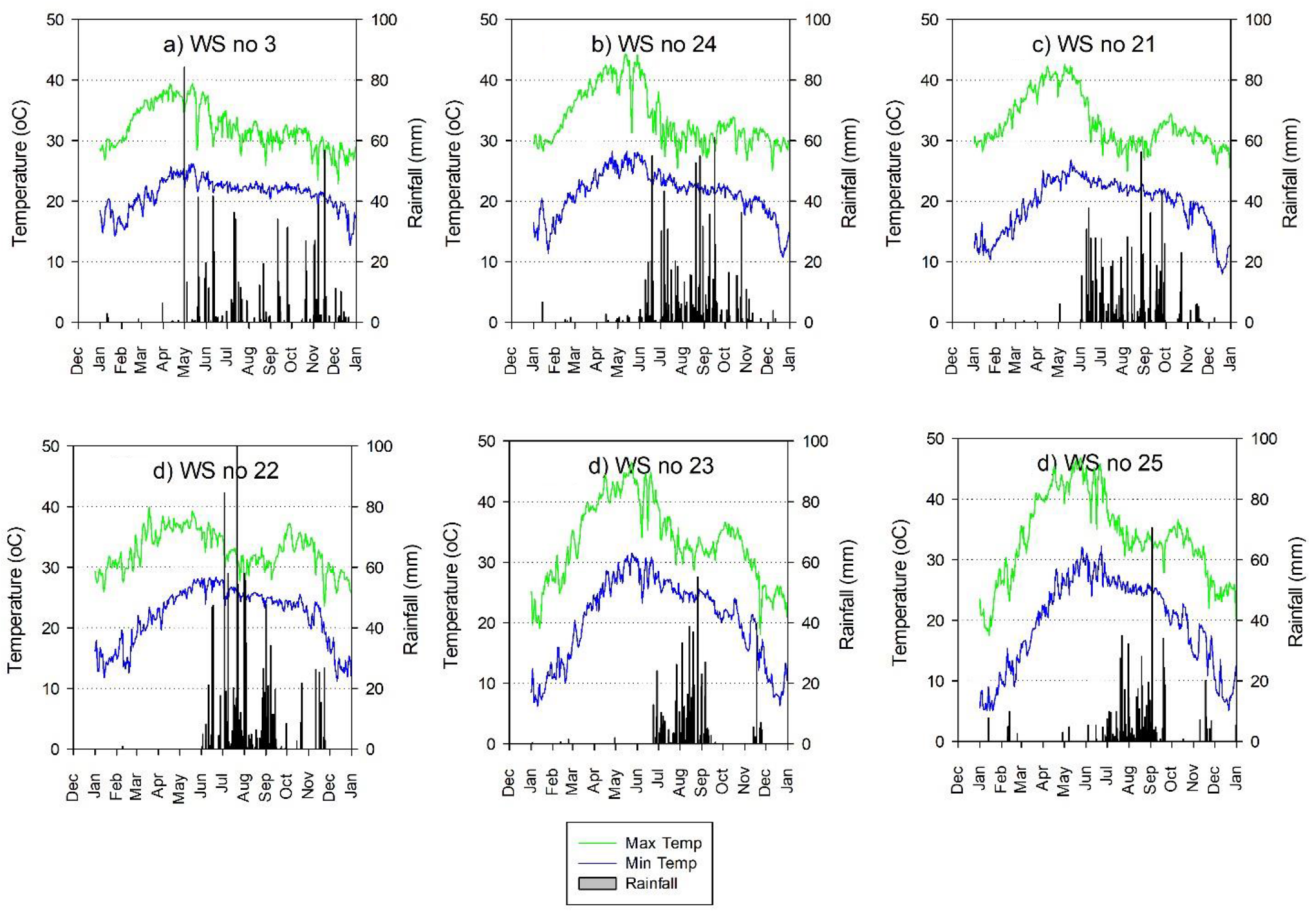
Variability in rainfall and maximum and minimum temperature in selected watershed locations in 2010.

Figure 5 shows the variability in temperature (maximum and minimum) and rainfall in 6 representative watershed locations (# 3, 21, 22, 23, 24, 25) on a daily time scale for all of year 2010. These watersheds represent the southern (# 3, 24), central (# 21, 25) and western (# 22, 23) semi-arid tropical regions. About 80–85% of the annual rainfall was concentrated in June and October. March, April and May are the hottest months with temperatures ranging between 35 and 45 °C. December and January are the coolest months with minimum temperatures ranging between 8 and 20 °C. While extreme temperatures were experienced mostly in the Central and Western semi-arid tropics, it ranged between 15 and 35 °C in Southern India for most of the period.

### Rainfall-runoff-soil loss relationship

**Figure 6:**
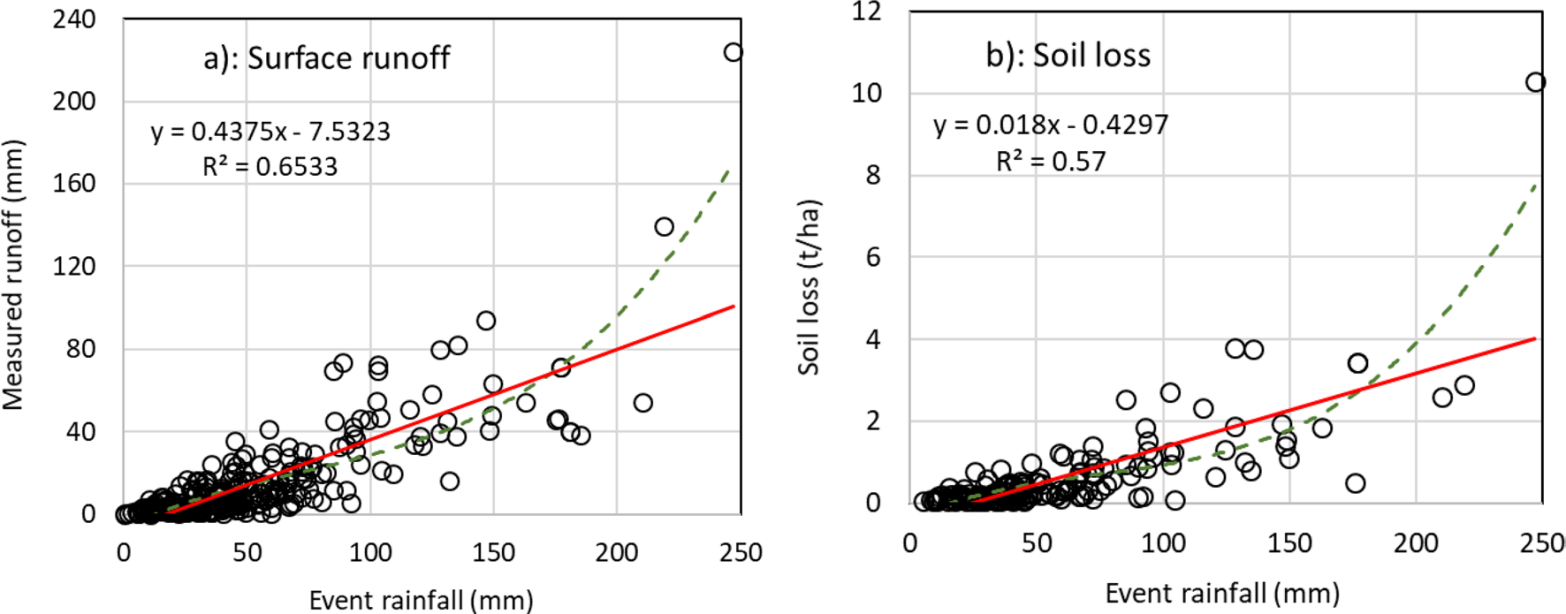
The relationship between rainfall events of different intensity (mm) and (**a**) runoff and (**b**) soil loss.

Figure 6 shows the relationship between rainfall events and runoff and soil loss from the different project locations. Rainfall intensity of up to 250 mm/day was recorded. Surface runoff generated varied by location depending on soil type and rainfall variability. A linear trend was observed in runoff generation up to 120 mm rainfall intensity which increased exponentially after 120 mm of rainfall. Average runoff at 120 mm rainfall was 40 mm (~ 33% runoff coefficient) compared to 230 mm (~ 90% runoff coefficient) for 250 mm/day of rainfall. Soil loss from rainfall events followed a similar pattern. Average soil loss was about 2 t/ha at 120 mm rainfall and 10 t/ha at 250 mm.

**Figure 7:**
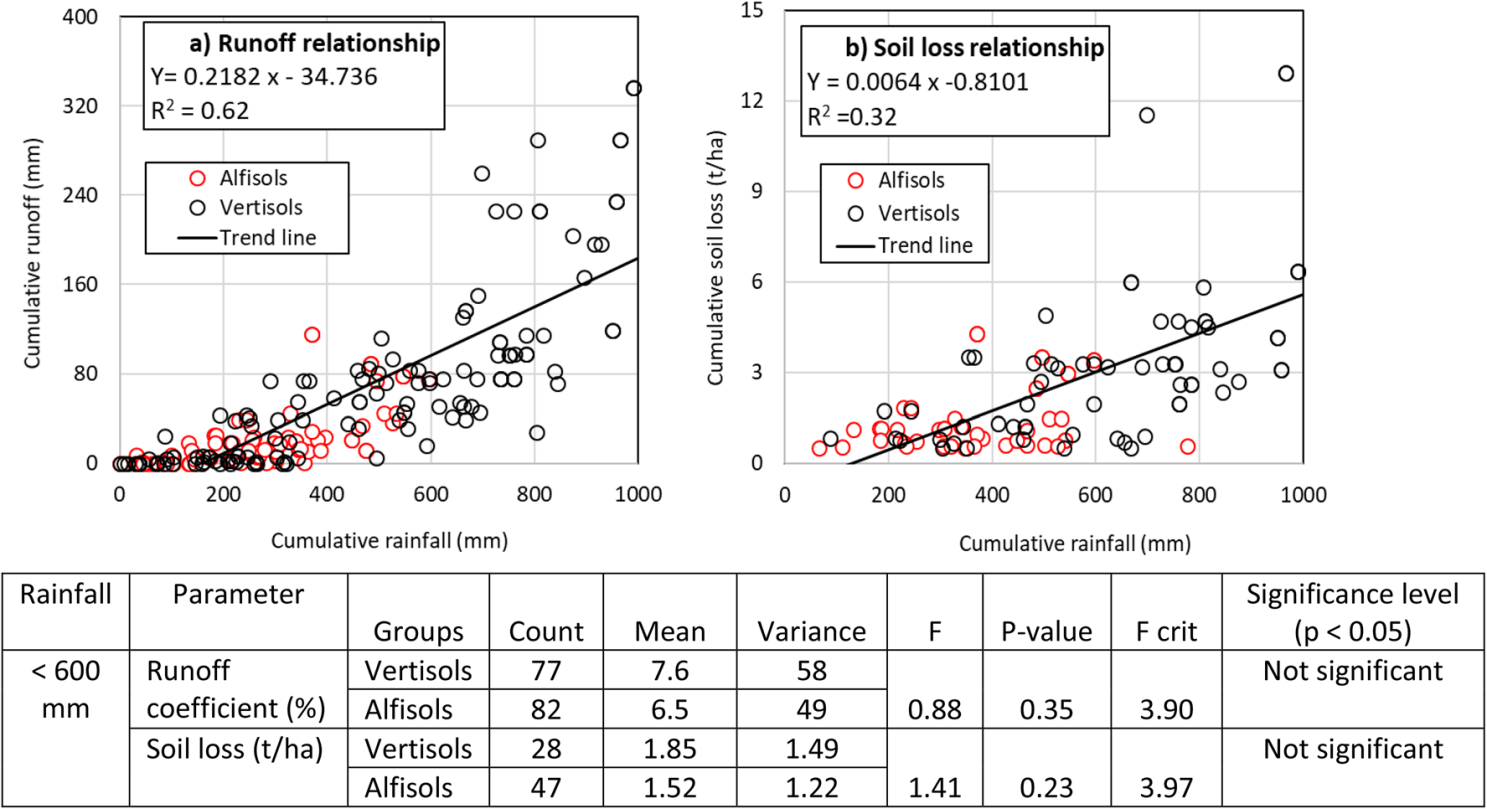
Relationship between cumulative rainfall and (**a**) cumulative runoff and (**b**) cumulative soil loss. The data is from 23 runoff and soil loss gauging stations in different watersheds. The red and black circles indicate responses in Alfisols and Vertisols, respectively.

Figure 7 describes the relationship between cumulative rainfall and runoff and soil loss in both Alfisols (red circles) and Vertisols (black circles) in the watersheds. Runoff below 200 mm cumulative rainfall was negligible. Runoff was between 20 and 80 mm for 200–600 mm of rainfall. The highest runoff recorded was 320 mm in response to 1000 mm of rainfall. Cumulative soil loss was found proportional to generated runoff. Soil loss was 0.1–3.0 t/ha for rainfall below 600 mm; whereas highest soil loss of 12 t/ha was recorded at 1000 mm rainfall. Alfisols-dominated watersheds received less than 600 mm rainfall whereas Vertisols-dominated watersheds received up to 1000 mm and more rainfall during the study period. The statistical results showed no significant difference in runoff coefficient (up to 600 mm rainfall) or soil loss in both Alfisols- and Vertisols-dominated watersheds.

**Figure 8:**
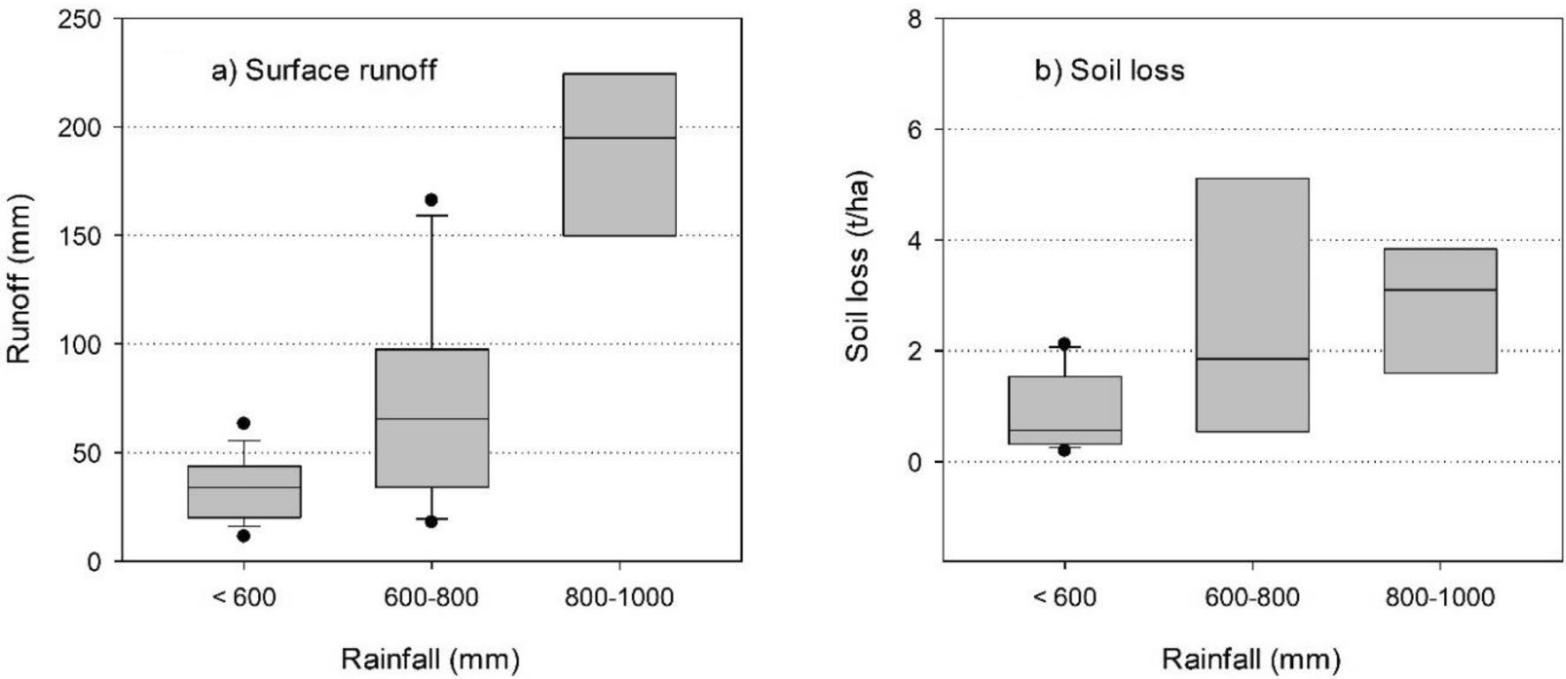
(**a**) Surface runoff and (**b**) soil loss from low, medium and high rainfall zones of the semi-arid tropics.

Rainfall-runoff relationship along with soil loss are summarized in low (< 600 mm), medium (600–800 mm) and high (800–1000 mm) rainfall zones in Fig. 8. Average runoff received in low, medium and high rainfall zones was 40 mm, 70 mm, and 190 mm, respectively. Soil loss in low, medium and high rainfall zones was 0.4 t/ha, 2.0 t/ha, and 3.3 t/ha, respectively.

Table 2 summarizes average annual surface runoff and soil loss along with rainfall in Alfisol- and Vertisol-dominated watersheds during the project period. In addition, peak intensity rainfall, runoff and soil loss measured in the watersheds sites are presented in Table 2. On an average, Alfisols watersheds were monitored for 2.5 years (ranging from 1 to 5 years) while it was 3.2 years (ranging from 1 to 7 years) in the Vertisols watersheds. Large variability was recorded from location to location in terms of cropping system and its management practices as these watersheds were spread across the country. In Alfisols watersheds, rainfall recorded ranged from 320 to 720 mm with mean of 524 mm while in the Vertisols watersheds 447 mm to 958 mm rainfall was recorded, with a mean rainfall of 717 mm. Average runoff coefficient in the Alfisols watersheds varied from 2 to 19% with a mean of 7.6% while it was between 3.5 and 24% in Vertisols watersheds, with a mean of 14%. Soil loss too varied; with average loss of 0.7 t/ha recorded in Alfisols watersheds and 2.6 t/ha in Vertisols watershed.

Peak rainfall intensity in Alfisols ranged from 25 to 210 mm/day, peak runoff was 5 mm/day to 71 mm/day and peak soil loss was 0.1 t/ha to 3.4 t/ha. In Vertisols-dominated watersheds, peak rainfall received varied from 32 to 247 mm/day, peak runoff was 5 mm/day to 224 mm/day and peak soil loss of 0.05 t/ha to 10.3 t/ha was recoded. The analysis of variance (ANOVA) indicated that rainfall received in both Alfisol- and Vertisol-dominated watersheds differed significantly, along with runoff and soil loss, as indicated by p-values in Table 2. It also indicated that peak rainfall events also differed significantly in Alfisol- and Vertisol-dominated watersheds. The runoff generated from high intensity events differed but soil loss was not significantly different. However, average soil loss recoded from high intensity events in Alfisols and Vertisols were 1.1 t/ha and 2.9 t/ha, respectively.

**Figure 9:**
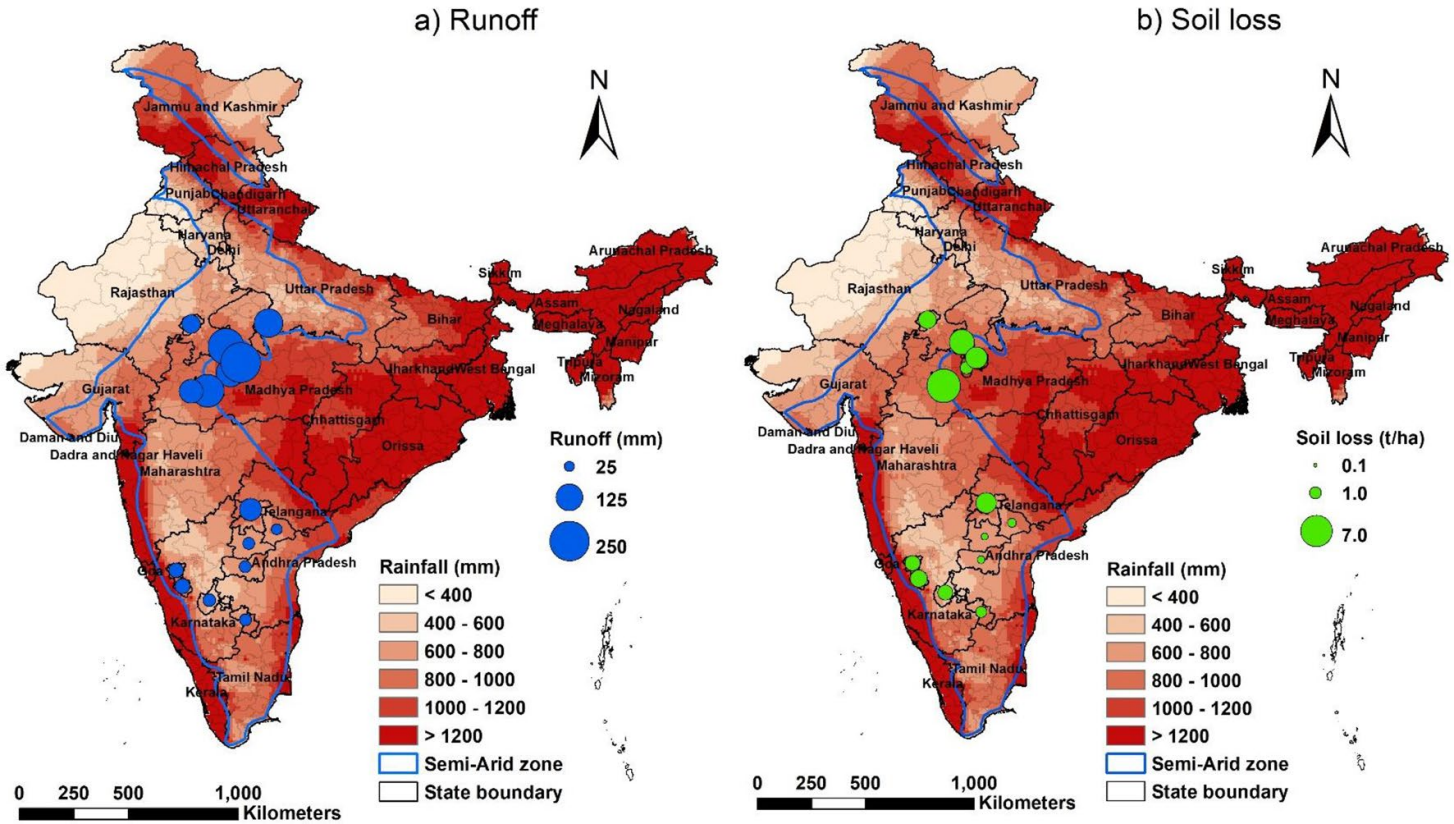
Spatial variability in (**a**) runoff and (**b**) soil loss in response to rainfall in different study watersheds.

Figure 9 shows the spatial variability in runoff generated and soil loss in the pilot watersheds. Average annual rainfall based on long-term gridded dataset (0.25°) between 1990 and 2018 was used as background layer. Runoff generated at watersheds located in southern states like Karnataka, Andhra Pradesh, and Telangana was less than 50 mm as rainfall in these areas was less than 600 mm during the monitoring years. On the contrary, watersheds located in central India (Madhya Pradesh and Uttar Pradesh) generated runoff between 100 and 250 mm in response to 800–1100 mm rainfall. Accordingly, soil loss varied from 2 to 7 t/ha.

**Figure 10:**
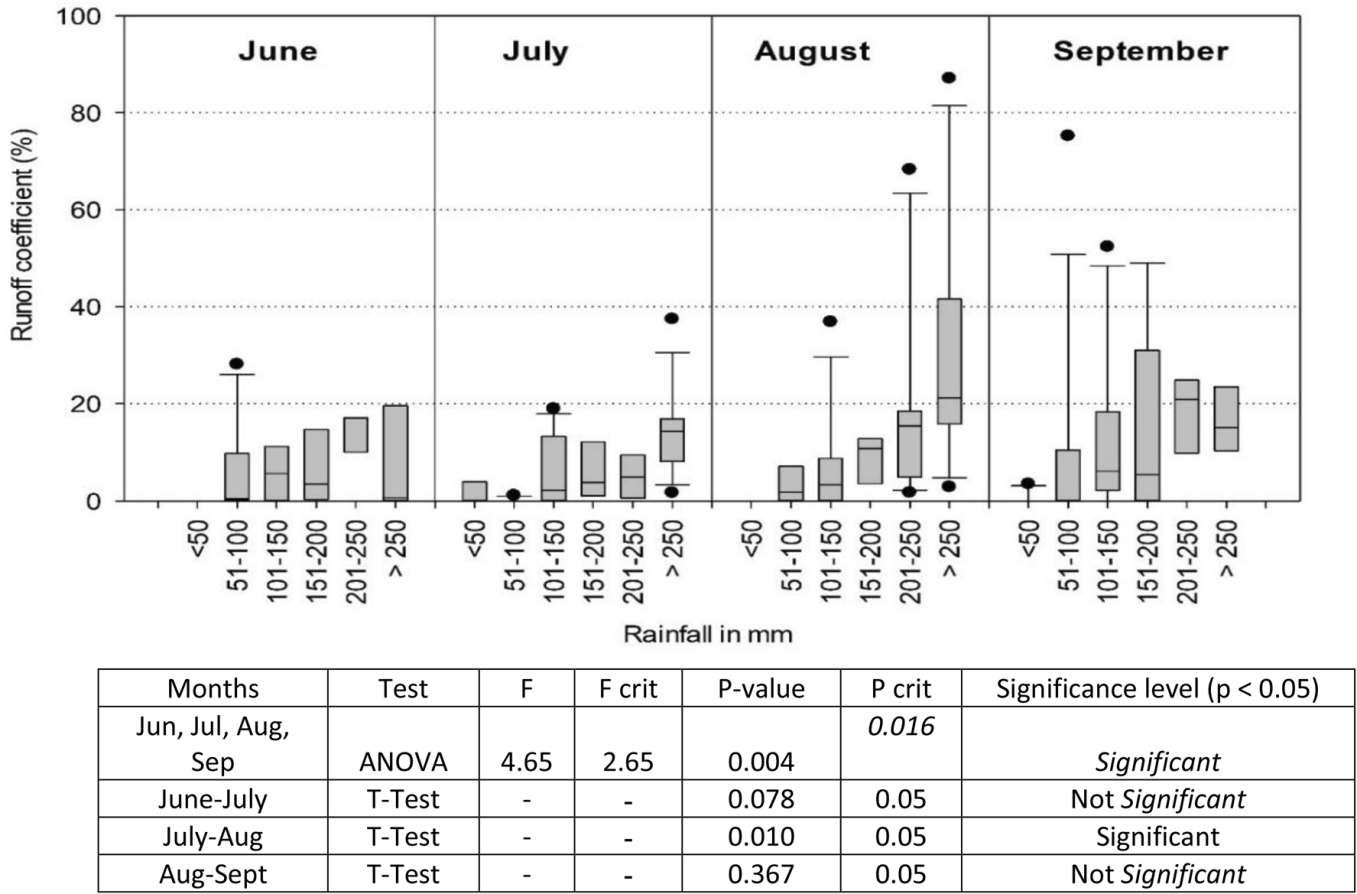
Runoff coefficients in different months with different amounts of rainfall every month.

Estimated runoff coefficients between June and September are given in Fig. 10. Total rainfall received in these months was split into six bands, differing by 50 mm intervals. Runoff coefficients for June were found to be less than 10%. March and May saw huge deficits in soil moisture as they coincided with the summer. Therefore, rainfall received in June and July was largely used to build moisture levels till saturation point was achieved to generate surface runoff. The month of August saw runoff generation and runoff coefficients increase proportionately with increased rainfall. However, a huge variability was observed in runoff coefficient due to rainfall intensity and available storage capacity. Rainfall during September started receding, therefore the runoff coefficients were found to be relatively lower than those in August. ANOVA analysis indicated significant differences in runoff coefficients in different months (p = 0.003). T-test showed no significant difference in runoff coefficients between June and July (p = 0.078) and significant difference between July and August (p = 0.01).

### Impact of rainwater harvesting interventions on runoff, soil loss, groundwater availability and crop intensification

**Figure 11:**
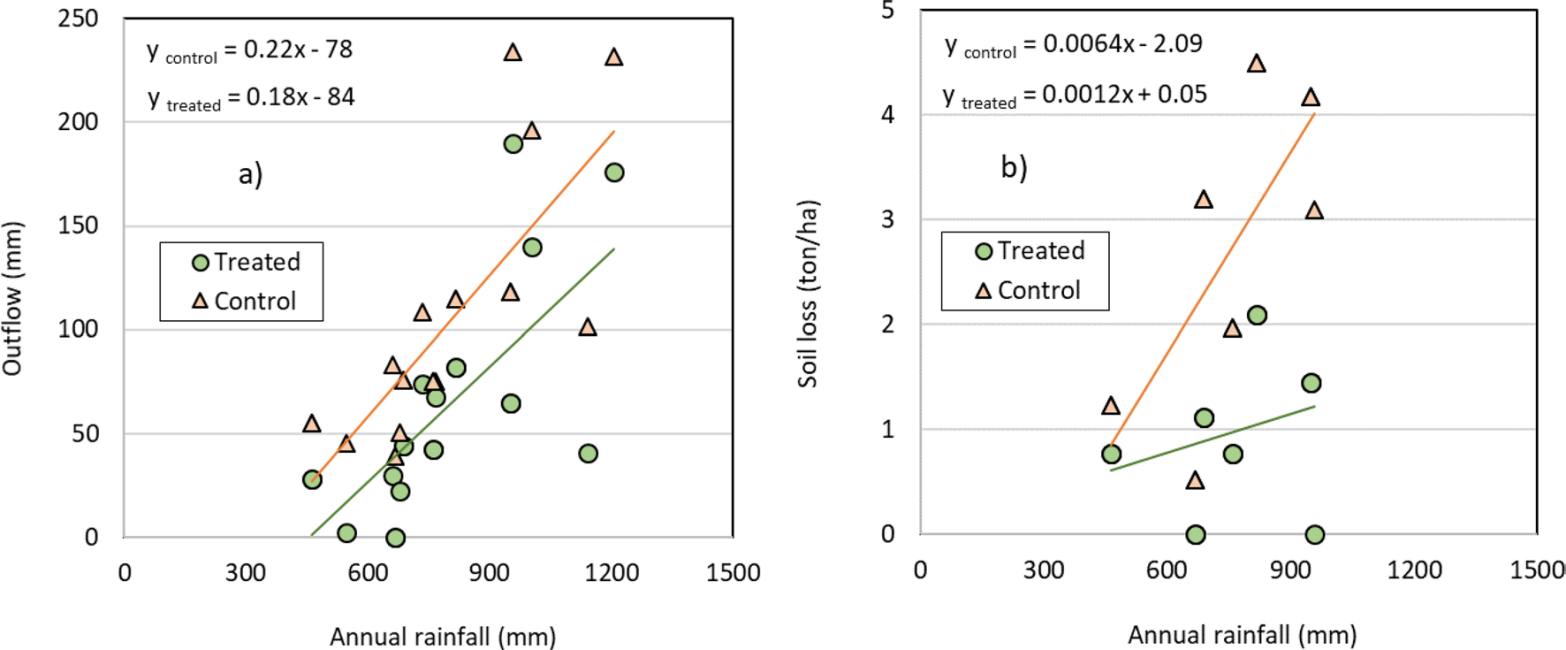
Reduction in (**a**) outflow and (**b**) soil loss measured following rainwater harvesting interventions in different rainfall zones.

Figure 11 describes the impact of rainwater harvesting interventions on outflow and soil loss. Rainwater harvesting interventions led to a runoff reduction of 25–60 mm compared to the control watersheds. For example, following the interventions, average outflow declined from 200 to 135 mm with 1200 mm rainfall. Similarly, outflow fell from 50 to 10 mm at 600 mm rainfall. The trendline of rainfall-runoff relationship in treated and control watersheds indicates that both the lines have followed a similar pattern, however with difference in magnitude (Fig. 11a). Due to various rainwater harvesting interventions, soil loss fell by 70% compared to that in control watersheds (Fig. 11b). With 900 mm of rainfall, soil loss dropped from 4 ton/ha to 1.2 ton/ha following RWH interventions (Fig. 11b). The trendline also clearly shows the decreasing pattern of soil loss in response to varying rainfall.

**Figure 12:**
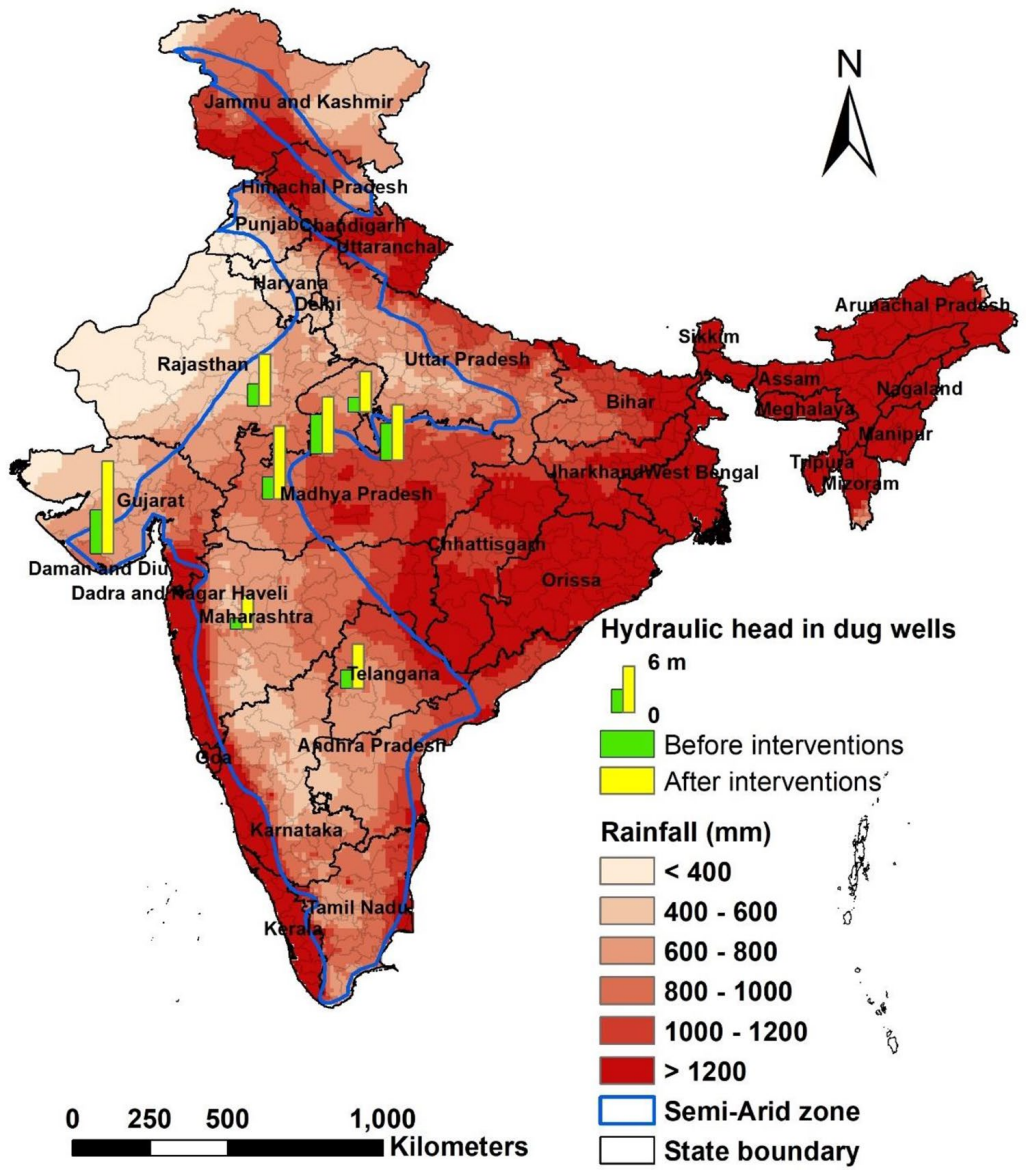
Impact of rainwater harvesting interventions on changes in groundwater status in October in benchmark watersheds.

Figure 12 shows the impact of rainwater harvesting interventions on groundwater availability after the monsoon (i.e., October) in eight benchmark watersheds. As October month is the representative month with highest groundwater level, therefore, data is presented for October month. The average hydraulic head of dug wells before and after the interventions are shown as green and yellow bars, respectively. The results show that on an average hydraulic head increased by 4 m (2.6–6.9 m) in dug wells during October due to rainwater harvesting interventions.

**Figure 13:**
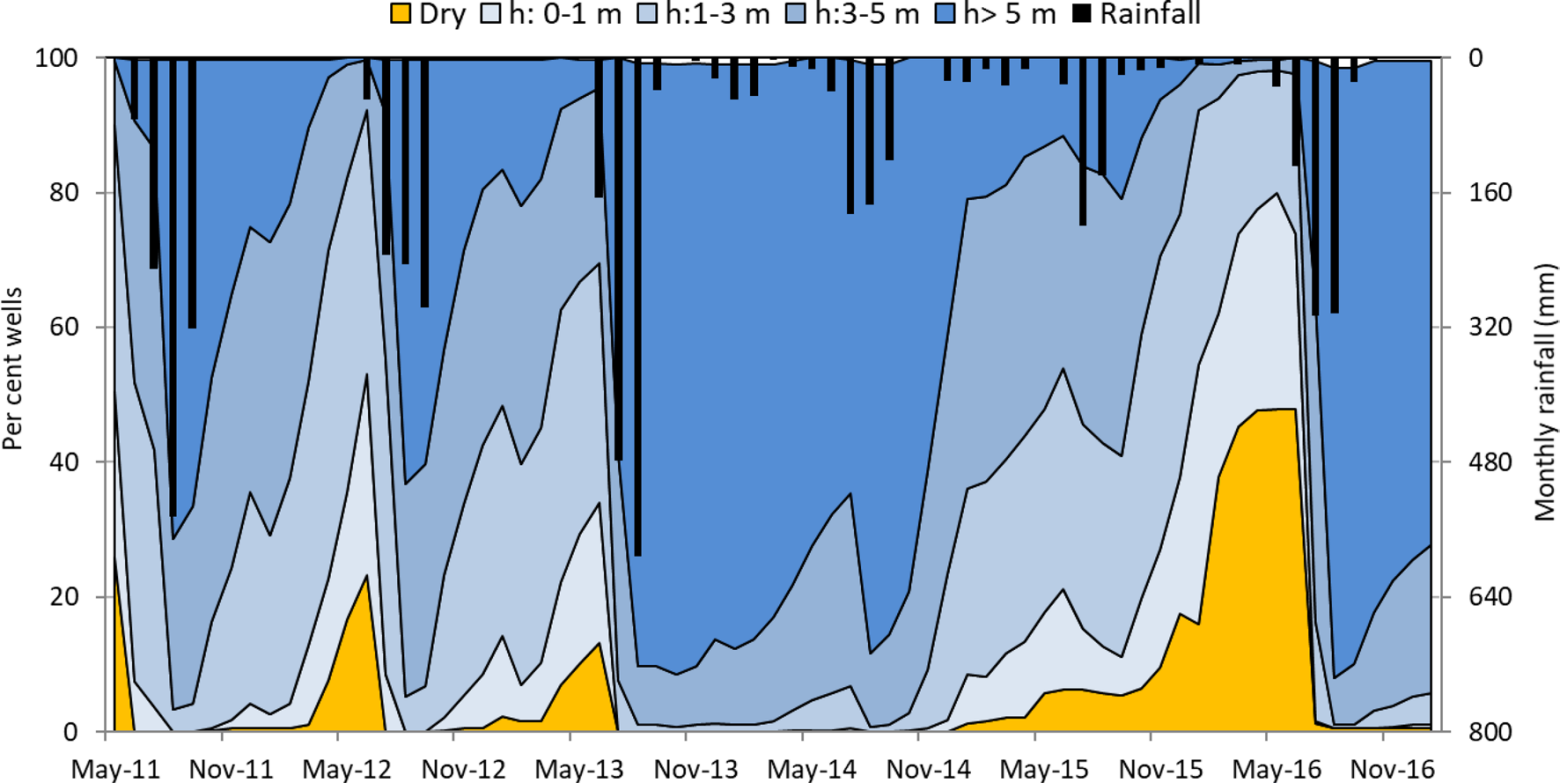
Data on the status of 388 dug wells in Parasai-Sindh watershed, Jhansi (watershed No. 25), monthly rainfall between 2011 (before interventions) and 2016; 2011 indicates before intervention status and 2013 indicates after project intervention status.

An example of the temporal variation in the functioning of dug wells in one of the benchmark watersheds (# 25) in Jhansi district, Central India, is shown in Fig. 13. Since the project was started in the year 2012, years 2011 and 2012 were considered as pre-intervention stages. The amount of rainfall received was 1189 mm in 2011, 1276 mm in 2013, 825 mm in 2012, 768 mm in 2016, 520 mm in 2014 and 404 mm in 2015. By 2013, over 70% of the rainwater harvesting structures were completed with 73,000 m^3^ storage capacity.

The dug wells were categorized into five groups based on pressure head: dry, poor (< 1 m), medium (1–3 m), good (3–5 m), and very good (> 5 m). A comparison of their functioning during 2011 and 2013 revealed that despite receiving similar amounts of rainfall, only about 60% and 25% of the dug wells showed very good status in July and December 2011 and 90% and 85% in August and December 2013, respectively. Once groundwater was recharged to its full potential in 2013, its availability was ensured in two consecutive dry years (i.e., up to December 2015). The wells started drying up only after December 2015 as it was one of the driest years (with 404 mm rainfall). A comparison of the two normal years (before 2012 and after 2016) showed similar results. For example, by the end of December 2012, the functioning of wells was very good in 19% of them, good in 38%, medium in 34% and poor/dry in 9% while the corresponding figures in December 2016 were 74%, 20%, 4% and 2%, respectively. This clearly shows that groundwater availability improved across the watershed villages during and after the interventions. Similar observations were noted in other benchmark locations. About 30–50% of the defunct wells have been brought back to functioning status and farmers were able to pump water for an additional 4 to 5 h every day. The well recharge recovery period improved significantly after the project interventions (Table 3).

**Table 3:** Impact of in-situ conservation and ex-situ rainwater harvesting interventions on various ecosystem services.

SN	Impact indicators	Before interventions			After interventions			No. of study watersheds
		Mean	Min	Max	Mean	Min	Max	
1	Rainfall (mm)	710	400	1390	710	400	1390	13
2	Runoff (mm)	100	30	320	60	5	300	13
3	Base flow period (days)	20	10	50	50	30	90	3
4	Soil loss (t/ha)	2.3	0.5	7.0	0.91	0.0	2.0	13
5	Hydraulic head in dug wells (m)							8
a	Monsoon	5	1	12	9	2	17	
b	Post-monsoon	2	1	3	6	4	10	
6	Functioning wells (%)	40	20	65	85	65	100	8
7	Pumping hours							6
a	Rainy season	4	3	6	9	5	11	
b	Postrainy season	2	1	3	6	2	7	
8	Recharge recovery period (h)							6
a	Rainy season	17	14	20	11	10	12	
b	Postrainy season	21	18	36	16	10	20	
9	Zone of influence (m)	–	–	–	600	150	1500	3
10	Land use (%)							6
a	Agriculture	54	40	70	73	65	95	
b	Waste/fallow	42	15	50	24	10	35	
c	Others	4	1	6	3	1	5	
i	Rainfed agriculture	45			20			
ii	Supplemental irrigation	5			28			
iii	Full irrigation	4			25			
11	Cropping intensity (%)	110	80	120	180	135	220	6
12	Crop yield (kg/ha)							20
a	Cotton							
b	Maize	3400	1300	4200	4200	3600	5000	
c	Groundnut	1100	880	1570	1450	950	2150	
d	Chickpea	1200	650	1500	2200	1800	2500	
e	Wheat	2100	1800	2400	3200	3000	3500	
13	Household income (US$/year)	800	600	1200	1800	1400	3500	6
14	Payback period (years)				3	2	4	3

Results further indicated that rainwater harvesting interventions increased base flow for an additional 30 days^43^. In addition, improved groundwater availability facilitated crop intensification, with 10–30% of the area which used to remain fallow being brought into productive cultivation^18^. Though cropping intensity and groundwater pumping improved, the functioning of wells was much better compared to pre-intervention status, which suggested groundwater resilience due to rainwater harvesting interventions. These interventions have spilled over to 1.5 km from the intervention areas^20^. A significant area (> 50%) under rainfed conditions was converted into either supplemental or full irrigation; this has directly impacted crop yields. Yields of cereals, pulses and oilseeds increased from 20 to 80% compared to the baseline stage. Total household income was influenced by increased area under cultivation and improved crop yields. Our economic analysis also showed that the payback period in these projects was less than 3 years^43^.

### Runoff probabilities and identifying potential zones for rainwater harvesting

**Figure 14:**
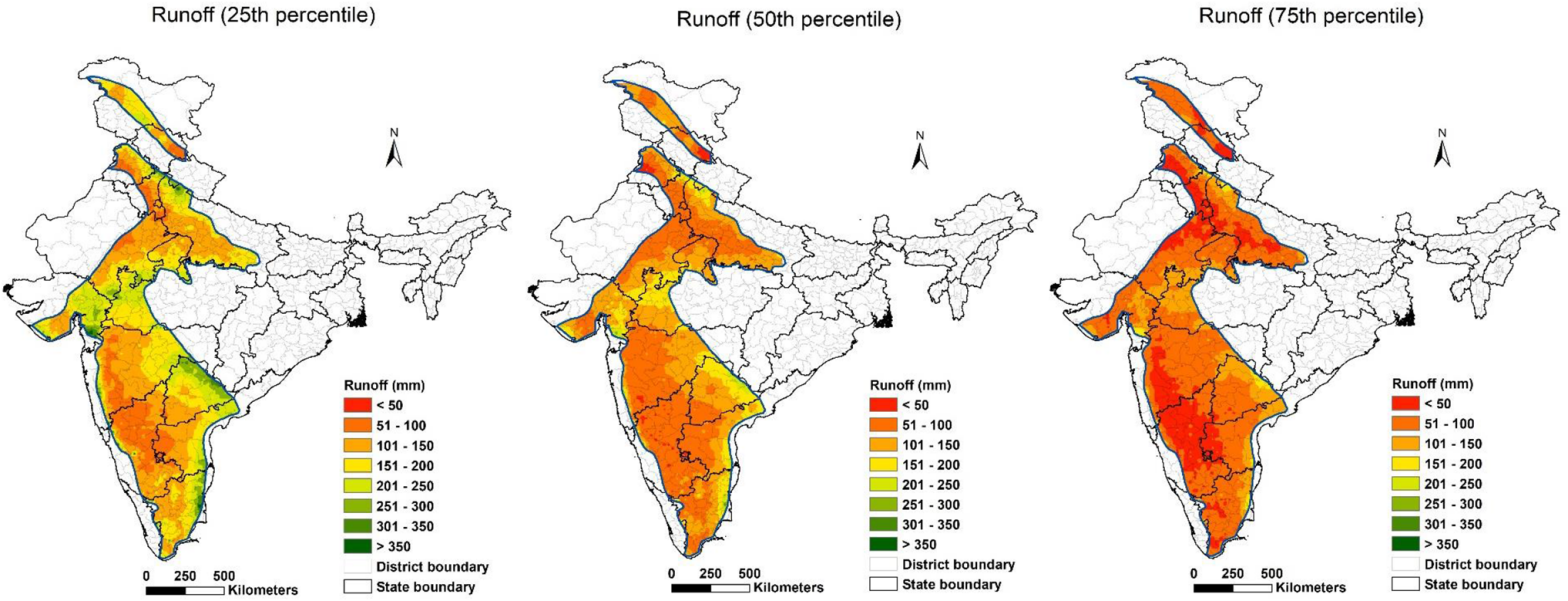
Runoff probabilities in the semi-arid tropics of the Indian subcontinent.

Figure 14 describes the probability of generating surface runoff at 25th, 50th and 75th percentiles across the Indian semi-arid tropics. A percentile is the value on a scale of one hundred that indicates the percentage of a distribution that is equal to or below it. In Fig. 14, the amount of runoff for a given grid point at 75th percentile was equal to or lower than 75% of the runoff values recorded among all years. Similarly, the 50th (median value) and 25th percentiles indicate that runoff amount was equal to or lower than 50% and 25% of the runoff values recorded, respectively. The higher the percentile, the lower is the runoff amount in the given timeframe.

In India, 128. 4 million ha fall under the semi-arid tropics, of which 22%, 58% and 20% of the area are likely to receive runoff of less than 50 mm; 50–100 mm, and 100–200 mm at 75th percentile. Further, 1%, 47%, 50% and 2% of the area are likely to get runoff of less than 50 mm, 50–100 mm, 100–200 mm, and > 200 mm at 50th percentile. Results at the 25th percentile indicate that 13%, 67%, and 20% of the area will be receiving runoff of 50–100 mm, 100–200 mm, and > 200 mm, respectively. The total estimated runoff for the semi-arid tropics in India at 75th, 50th, and 25th percentiles is estimated to be 95 billion cubic meter (BCM), 138 BCM and 200 BCM, respectively. The Indian semi-arid tropics experience large variability in runoff generation. Regions that experienced more than 200 mm runoff (median values indicated in the 50th percentile) also experienced low runoff (indicated in the 75th percentile probability) due to annual variability in rainfall.

**Figure 15:**
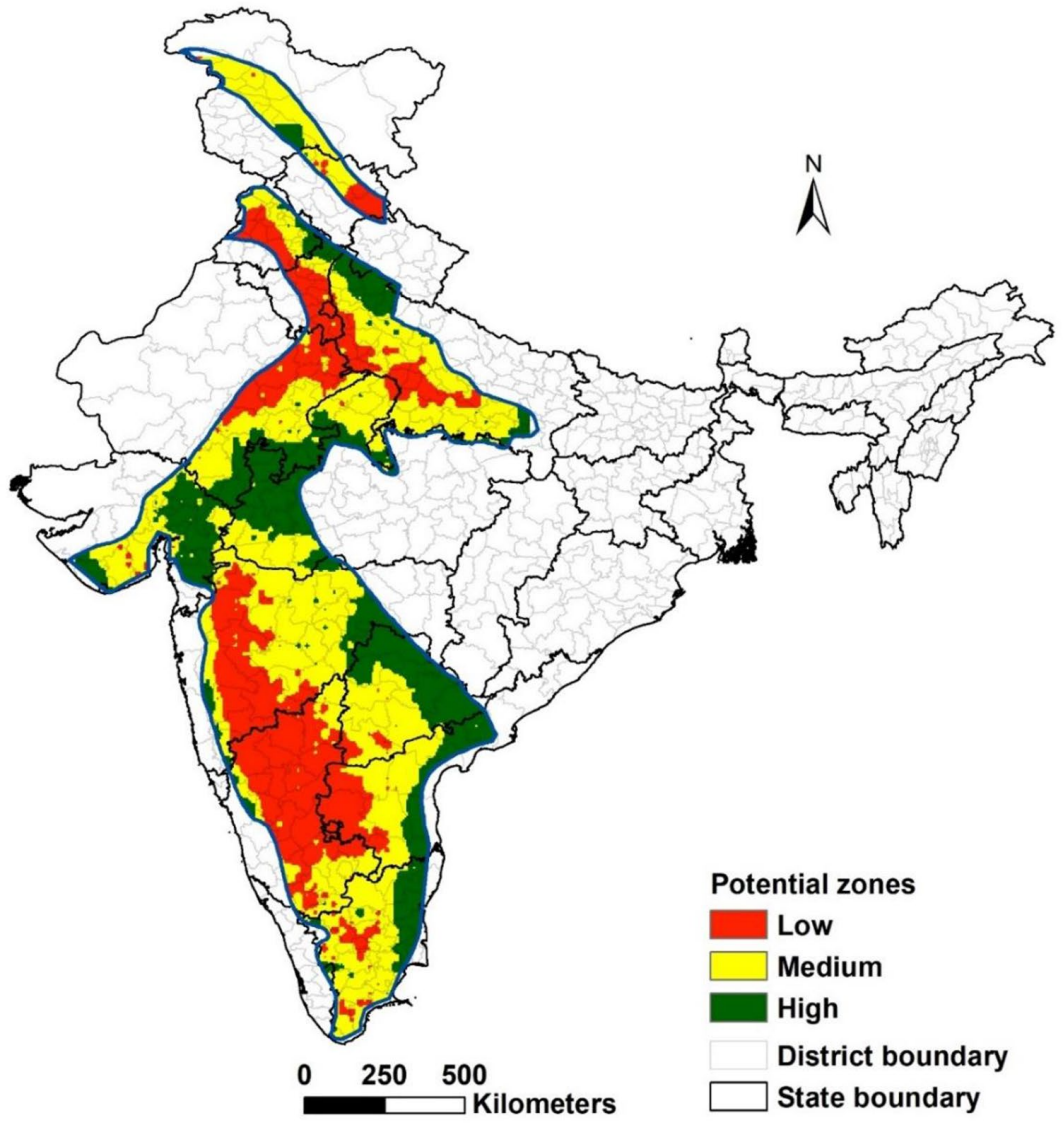
Potential zones to undertake rainwater harvesting interventions in the semi-arid tropics of India.

Figure 15 identifies potential zones for undertaking rainwater harvesting interventions based on runoff at the 50th percentile. Results showed that of the total 128.4 million ha under semi-arid tropics, 24% and 49% of the area were high and medium potential zones and 27% were low potential zones. In high and medium potential zones, surplus runoff availability was more than 70% and 50%, respectively, despite RWH interventions. Surplus runoff may decline by more than 50% in low potential zones compared to non-intervention status. The analysis showed that Madhya Pradesh, Central Maharashtra, Telangana, Central and Eastern Andhra Pradesh, and Uttar Pradesh hold immense potential for rainwater harvesting interventions as more than 70% of the semi-arid tropical area in these states generates runoff ranging between 150 and 250 mm. Harvesting a portion of this (~ 40 mm) for upland development will result in marginally reducing water to downstream users. States like Karnataka, part of Maharashtra, Western Andhra Pradesh, parts of Punjab, Haryana, and Rajasthan have low water harvesting potential as they face physical water scarcity. However, the 25th percentile runoff analysis showed that these zones also get more than 100 mm runoff (once in four year) and hence hold potential for undertaking rainwater harvesting interventions. It is also to be noted that this analysis did not take into account other land uses such as wastelands and slopes that also generate significant surface runoff; hence site-specific decisions must be made based on topography and land use characteristics.

### Uncertainty in the analysis

The current analysis was largely based on rainfall and runoff relationships in the agricultural landscape. However, there is a huge diversity in land use in different parts of the semi-arid tropics. In addition, all the monitoring stations captured the hydrology of a mild slope (up to 2%) and did not capture runoff in higher slopes. Data in the watersheds were monitored for a short period. Of the 25 watersheds, 4 watersheds were monitored for 5–7 years, and the average monitoring period of the study watersheds was 3 years. While this may have captured hydrology partially and these relationships can be improved with long-term data monitoring. In addition, the scale of monitoring also makes a difference. Of the 25 watersheds, only 6 monitored were of 300–1000 ha and others were of 10–30 ha. This is also important to analyse the impact of scale on water balance components in future research. In addition, monitoring soil moisture and simulation modeling are required to understand all the water balance components including groundwater recharge, actual evapotranspiration, and soil moisture change and its links with land use and cropping systems.

## Discussion

There are opportunities for rainwater harvesting interventions in regions where rainfall is higher than 600 mm. A multilocation analysis has shown that on average 40 mm of runoff/year is harvested additionally at upstream locations due to various rainwater harvesting interventions. This significantly contributes to landscape restoration and strengthens ecosystem services and crop intensification. The semi-arid tropics generally have 30–50 rainy days in a year and these ecologies are largely rainfed or supported by groundwater resources^47^. Rainwater harvesting interventions help enhance groundwater recharge to make it available for a longer period^18^, failing which there is huge uncertainty of resource availability due to variability in rainfall and consequent risk of crop failure. A significant amount of residual soil moisture in current rainfed systems is lost to non-productive evaporation either during the rainy or postrainy season if left uncultivated^48^. In the absence of supplemental irrigation, farmers prefer to cultivate their lands in any one season (either rainy or postrainy) as there are otherwise chances of crop failure^10^. Nearly 30–40% of the cultivable area is left fallow in either seasons in Uttar Pradesh, Madhya Pradesh, and Chhattisgarh due to water scarcity despite rainfall ranging from 800 to 1100 mm/year^49^. The uplands in these regions experience land degradation and acute water scarcity in addition to food insecurity, poverty and malnutrition^50,51^. At the same time, downstream areas encounter floods and waterlogging. The introduction and adoption of both resource augmentation and conservation technologies promise to transform such degraded ecologies. With the availability of supplemental irrigation, these systems hold huge potential to be converted from subsistence to surplus production systems without exerting extra pressure on available resources^52,53^.

Given that many parts of the country are nearing absolute (physical) water scarcity, there is limited scope for sustainable out-take of surface or groundwater. This calls for a closer examination of rainfed systems’ production and productivity *vis-a-vis* supplemental or full scale irrigation investments for local and national food security. Our results demonstrated the huge untapped potential to build system level resilience in dryland ecologies. Since rainfed systems are subject to more rainfall variability compared to fully irrigated systems, the focus should be on conservation and supplemental irrigation to explore the unrealized potential of rainfed systems.

The scarcity of freshwater in the semi-arid tropics underlines the need for rainwater harvesting interventions that may result in developing trade-offs between upstream and downstream users^18,19^. However, these trade-offs are not always negative^54,55^. Our analysis shows that rainwater harvesting interventions may reduce outflow by 30–50% in regions with 600–800 mm rainfall and by less than 30% in regions with more than 800 mm rainfall. At the same time, they are helpful in controlling floods and soil loss^19,56^. In wet years, rainwater harvesting interventions marginally harvest water in the uplands without negatively affecting freshwater availability for downstream users as surplus is very high^18,43^. On the contrary, this freshwater availability in dry years is insufficient for both upstream and downstream users due to the physical water scarcity faced in such years. Thus, the greater concern is for average years with moderate rainfall. Marginal harvesting of freshwater has been known to transform these landscapes from degraded to productive ecosystems^17–19,21,43^.

Findings of this study provide insights to stakeholders and donors to design and prioritize investments in designing and implementing various land and water management interventions. Learnings from the study can also be replicated in other countries in the semi-arid tropics of Asia and Africa that face similar challenges.
